# Response of *Amaranthus* Species to Co-Application of Cattle Manure Microdoses, Mineral Fertiliser, and Arbuscular Mycorrhizal Fungi Inoculation on Acidic Soils in South Africa

**DOI:** 10.3390/plants15030441

**Published:** 2026-01-31

**Authors:** Simphiwe Mhlontlo, Tafadzwanashe Mabhaudhi, Nqaba Nongqwenga, Tembakazi Theodora Silwana, Mpaballeng Alinah Ramangoele, Bongani Petros Kubheka, Pardon Muchaonyerwa

**Affiliations:** 1 Döhne Agricultural Development Institute, Private Bag X15, Stutterheim 4930, Eastern Cape, South Africa; tembakazi.silwana@ecagriculture.gov.za (T.T.S.); alinah.ramangoele@ecagriculture.gov.za (M.A.R.); kubhekab@arc.agric.za (B.P.K.); 2 School of Agriculture and Science, University of KwaZulu-Natal, Private Bag X 01, Scottsville, Pietermaritzburg 3201, KwaZulu-Natal, South Africa; mabhaudhi@ukzn.ac.za (T.M.); muchaonyerwa@ukzn.ac.za (P.M.); 3 Centre for Transformative Agricultural and Food Systems, School of Agricultural, Earth and Environmental Sciences, University of KwaZulu-Natal, Private Bag X01, Scottsville, Pietermaritzburg 3201, KwaZulu-Natal, South Africa; 4 Agricultural Research Council, Plant Health & Protection, R573 Moloto Road, Roodeplaat, Pretoria 0102, Gauteng, South Africa

**Keywords:** *Amaranthus*, acidic soil, cattle manure, mineral fertiliser, arbuscular mycorrhizal fungi

## Abstract

Low soil nutrient availability and uptake negatively affect crop productivity in acidic soils. For example, phosphorus (P) availability is reduced by fixation of aluminium (Al) and iron (Fe) hydrous oxides and precipitation with soluble Al and Fe. In addition, soil acidity inhibits root growth, and application of agricultural lime ameliorates these challenges, thereby improving yields. However, resource-limited farmers in the Eastern Cape Province can rarely afford to procure lime and chemical fertilisers, which necessitates alternative approaches to addressing the challenge of low nutrient availability for crops. The present study explores interactions between cattle manure and mineral fertiliser applications coupled with arbuscular mycorrhizal fungi (AMF) inoculation on the agronomic performance of *Amaranthus* grown in acidic soil. The treatments were 100% cattle manure, 50% cattle manure + 50% NPK fertiliser and lime, 33% cattle manure + 33% NPK and lime + AMF, the recommended rate of mineral fertiliser and lime, AMF, and an absolute control. Cattle manure and mineral fertiliser application, including mixtures of their microdoses, coupled with AMF inoculation, significantly improved the growth and yield of *Amaranthus* species. Leaf tissue concentrations of N, P, K, Ca, Mg and Zn and their uptake, and selected residual soil properties and nutrients increased significantly following application of the treatments relative to the unfertilised control. The findings of this study imply that application of manure and mixtures of microdoses and mineral fertiliser, together with AMF, improve nutrient uptake and yield of *Amaranthus* and residual nutrients that benefit subsequent crops.

## 1. Introduction

Climate change in South Africa has been observed to have low and erratic rainfall patterns [1]. This condition has exacerbated water scarcity challenges, ultimately threatening food security [2]. According to Taruvinga et al. [3], promoting the consumption of indigenous, nutritious plant species such as *Amaranthus* spp., which have low moisture and external input requirements, could be an effective adaptation strategy to curb poverty and malnutrition.

*Amaranthus* spp. are indigenous leafy vegetables well adapted to prevailing climatic conditions compared to introduced leafy vegetables [4]. Mabhaudhi et al. [1] highlighted that most indigenous plants possess traits that make them ideal for production in areas with marginal conditions.

According to [4], *Amaranthus* spp. belong to the *Amaranthaceae* family and the genus *Amaranthus,* which comprises about 60 species, of which 40 are native to America, while the rest are endemic to Africa, Asia, and Europe. Zhigila et al. [5] earlier declared that about seventeen *Amaranthus* spp. are cultivated and consumed as leafy vegetables worldwide, while three are classified as grain types. Although *Amaranthus* spp. are recognised as low-management crops that thrive under marginal soil and environmental conditions, Dlamini et al. [6] reported that their yield could be improved through appropriate fertilisation.

Crop production in most regions of the Eastern Cape Province, South Africa, is challenged by soil acidity, with a pH below 5.0 due to high rainfall exceeding 600 mm per annum [7]. Accumulation of soluble aluminium (Al) and iron (Fe) in acidic soils inhibits root growth, while their precipitation with phosphorus (P) and P fixation on their hydrous oxides reduces P uptake [8,9].

The application of mineral fertilisers and agricultural lime has long been recognised as ameliorating the adverse effects of soil acidity and improving crop yields [10]. However, resource-constrained farmers in the affected areas apply animal manure to their fields to address declining soil fertility [11]. When animal manures are applied to acidic soils, the variety of organic acids that decompose form stable complexes with Al and Fe, blocking P retention sites, and making P more available to plants [12].

Another alternative for overcoming challenges associated with the loss of plant nutrients in resource-limited environments is the introduction of arbuscular mycorrhizal fungi (AMF) [13]. According to Othman et al. [14], AMF can be applied as an inoculant to seeds, roots, and growing media to enhance crop growth and yield whilst mitigating the negative impacts caused by abiotic stresses such as drought, heat, and soil acidity. Clark and Zeto [15] reported that AMF colonise the plant’s root system and form a network of fine filaments that associate with plant roots and draw nutrients and water from the soil that the root system would not be able to access otherwise.

These soil microorganisms have been utilised to stimulate plant growth and improve yields under both biotic and abiotic limiting conditions [16]. They increase the root surface area and ultimately improve water and nutrient uptake. Jangandi et al. [17] observed improved plant height, biomass, and yield of *Amaranthus paniculatus* inoculated with a combination of AMF, *Azotobacter*, and phosphate-solubilising bacteria (PSB). Jangandi et al. [17] further emphasised that microorganisms enhance the uptake of phosphorus and nitrogen (N), improve soil structure, and increase stress tolerance in plants, which ultimately reduces reliance on chemical fertilisers for sustainable leafy vegetable production.

Nonetheless, there is a paucity of research exploring the synergistic effects of animal manures, the co-application of microdoses of manure and mineral fertilisers, and AMF inoculation in crop production on the acidic soils of the Eastern Cape. Hence, this study aimed to assess the effects of cattle manure as a sole organic fertiliser, as well as co-application of mixtures in different proportions with NPK fertilisers, coupled with AMF inoculation, on growth, nutrient uptake, *Amaranthus* yield, and post-harvest soil properties.

## 2. Results

### 2.1. Effects of Cattle Manure–Mineral Fertiliser Co-Fertilisation, and AMF Inoculation on Growth Attributes of Amaranthus

Highly significant differences (*p* < 0.01) were observed in plant height, number of leaves, stem girth, and root length due to the co-application of cattle manure and mineral fertiliser coupled with AMF inoculation across the cropping seasons (Table 1).

#### 2.1.1. Plant Height

In both cropping seasons, the fertilised treatments produced the tallest plants relative to both unfertilised and AMF-inoculated controls. In the first cropping season, mineral fertilisation produced the tallest plants (74.39 cm), outcompeting 100% cattle manure (CM) (61.36 cm), ½ CM + ½ RFL (65.23 cm), and ⅓ CM + ⅓ RFL + AMF (66.05 cm), which were comparable. In the preceding cropping season, fertilisation with 100% cattle manure (CM) resulted in the tallest plants (50.57 cm), which were comparable to mineral fertilisation (RFL) (47.46 cm). Application of ½ CM + ½ RFL and ⅓ CM + ⅓ RFL + AMF resulted in comparable plant heights of 42.35 cm and 36.69 cm, respectively. The shortest plants (30.72 cm) in the preceding season were attained from the unfertilised control (C) and were comparable to the inoculated control (AMF) (31.61 cm), which was similar to the succeeding cropping season, where both unfertilised and inoculated controls yielded the shortest plants of 24.95 cm and 25.63 cm, respectively. No significant differences were observed for the plant heights of *A. hybridus* L. and *A. cruentus* species in Season 1, while *A. hybridus* produced taller plants (44.74 cm) than *A. cruentus* (31.15 cm) in the succeeding season.

#### 2.1.2. Number of Leaves

In the first season, plants fertilised with mineral fertiliser (RF) produced the highest number of leaves (93.00), comparable to ⅓ CM + ⅓ RFL + AMF (82.00). Plants dosed with 100% CM, and those dosed with ½ CM + ½ RFL, generated an equivalent number of leaves (75.12 and 75.65, respectively) while the least and comparable number of leaves (26.42 and 27.69) was attained from both unfertilised and inoculated controls.

All fertilised plants in the succeeding cropping season yielded plants with comparable numbers of leaves, ranging from 36.17 to 46.25, while unfertilised and AMF-inoculated controls formed the lowest number of leaves (21.83 and 22.85, respectively). *A. hybridus* produced the highest number of leaves in both seasons: 87.73 in the first season and 48.86 in the following season. This was compared to *A. cruentus* with 38.91 in the first season and 22.80 in the subsequent season.

#### 2.1.3. Stem Girth

Plants fertilised with mineral fertiliser produced the thickest stems (15.33 cm) in the first cropping season and were comparable to ⅓ CM + ⅓ RFL + AMF (13.96 cm), outcompeting ½ CM + ½ RFL (13.42 cm) and 100% CM (12.30 cm). Inoculated (AMF) and absolute controls produced the thinnest stems (5.35 cm and 5.74 cm, respectively). In the subsequent cropping season, fertilisation with RFL, CM, and ⅓ CM + ⅓ RFL + AMF resulted in the thickest and comparable stem girths of 9.41 cm, 9.23 cm, and 8.16 cm, respectively, outdoing the ⅓ CM + ⅓ RFL + AMF mixture (7.06 cm). Similar to the first season, the thinnest plant stems in the second cropping season were attained from both unfertilised and AMF-inoculated controls (4.87 cm and 5.16 cm, respectively). No significant difference was observed in the stem girth of *Amaranthus* species emanating from fertilisation and AMF inoculation in both seasons.

#### 2.1.4. Root Length

In both cropping seasons, plants subjected to different treatments and combinations produced longer roots relative to unfertilised and AMF-inoculated controls. In the initial cropping season, plants exposed to various treatments produced the longest and comparable roots, ranging from 17.42 cm to 18.32 cm, and the shortest roots (11.81 cm) were observed in the unfertilised control, which was comparable to the inoculated control (12.15 cm). Even though plants fertilised with 100% CM in the second cropping season produced the longest roots (33.33 cm), they were comparable to ⅓ CM + ⅓ RFL + AMF (12.78 cm), RFL (12.73 cm), and ½ CM + ½ RFL (12.13 cm). Unfertilised and AMF-inoculated controls generated the shortest roots, 9.34 cm and 9.85 cm, respectively. No significant difference was observed in the root length of *Amaranthus* species emanating from fertilisation and AMF inoculation in both seasons.

### 2.2. Effects of Cattle Manure–Mineral Fertiliser Co-Fertilisation, and AMF Inoculation on Yield Attributes of Amaranthus

**Figure 1:**
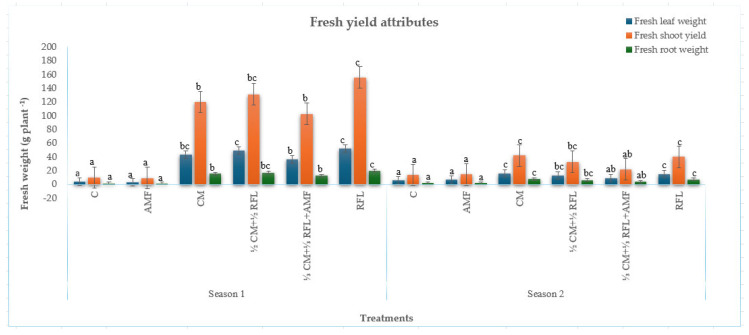
Effects of cattle manure–mineral fertiliser co-fertilisation and AMF inoculation on fresh yield attributes of *Amaranthus.* Different superscript letters (a, b, and c) within a column indicate differences between treatment combinations.

**Figure 2:**
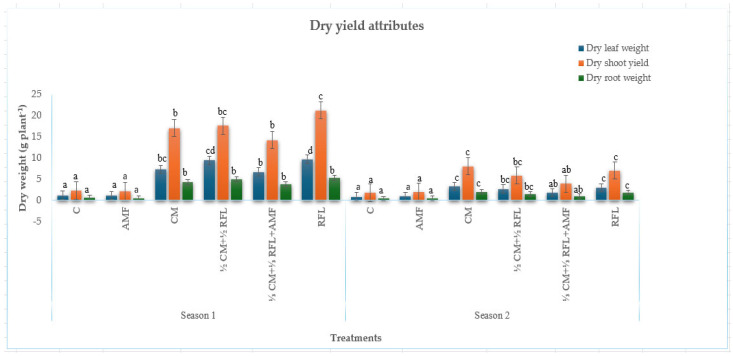
Effects of cattle manure–mineral fertiliser co-fertilisation and AMF inoculation on dry yield attributes of *Amaranthus.* Different superscript letters (a, b, and c) within a column indicate differences between treatment combinations.

Application of cattle manure, NPK fertiliser, and inoculation with AMF resulted in highly significant increases (*p* < 0.001) in the fresh (Figure 1) and dry (Figure 2) weight of *Amaranthus*, relative to the unfertilised and AMF-inoculated controls, in both growing seasons.

#### 2.2.1. Fresh Weight

##### Fresh Weight of Leaves

In the first cropping season, plants subjected to mineral fertilisation generated the highest fresh weight of leaves (52.50 g/plant) and were equivalent to ½ CM + ½ RFL fertilisation (49.65 g/plant), outperforming 100% CM (43.59 g/plant) and ⅓ CM + ⅓ RFL + AMF (36.74 cm). The least fresh weight of leaves was attained from both the unfertilised control (4.55 g/plant) and AMF-inoculated control (3.43 g/plant). In the second cropping season, the highest fresh leaf weight was observed with 100% CM (15.99 g/plant), which was equivalent to RFL (15.06 g/plant). Fertilisation with ½ CM + ½ RFL, and ⅓ CM + ⅓ RFL + AMF produced comparable fresh leaf weight (13.19 g/plant and 9.26 g/plant, respectively), while unfertilised and AMF-inoculated controls produced the lowest weight (6.29 g/plant and 6.61 g/plant, respectively).

##### Fresh Shoot Yield

The heaviest shoot weight (155.95 g/plant) in the first cropping season was recorded following recommended mineral fertilisation (RFL) and was comparable to ½ CM + ½ RFL fertilisation (131.29 g/plant). Plants exposed to 100% CM and ⅓ CM + ⅓ RFL + AMF produced equivalent shoot weights (120.00 g/plant and 102.78 g/plant, respectively), while both unfertilised and AMF-inoculated controls recorded the lowest weights (10.11 g/plant and 9.40 g/plant, respectively).

In the subsequent cropping season, mineral fertilisation also produced the highest shoot weight (40.13 g/plant), which was comparable to 100% CM fertilisation (41.99 g/plant) and ½ CM + ½ RFL fertilisation (32.98 g/plant), while the lowest weight was attained from both unfertilised and inoculated controls (14.02 g/plant and 14.64 g/plant, respectively).

##### Fresh Root Weight

Recommended mineral fertilisation (RFL) recorded the highest fresh root weight (20.28 g/plant) in the first cropping season, compared to ½ CM + ½ RFL fertilisation (17.16 g/plant). Plants fertilised with 100% CM and ⅓ CM + ⅓ RFL + AMF produced comparable root weights (15.56 g/plant and 12.53 g/plant, respectively), while the lowest weight was attained from both unfertilised and inoculated controls (1.60 g/plant and 1.43 g/plant, respectively). In the following season, 100% CM fertilisation yielded the highest fresh root weight (7.78 g/plant), which was comparable to mineral fertilisation (7.30 g/plant) and ½ CM + ½ RFL (6.18 g/plant), and outcompeted ⅓ CM + ⅓ RFL + AMF (3.66 g/plant). The lowest weight was recorded in both the unfertilised and inoculated controls (1.91 g/plant and 2.05 g/plant, respectively).

#### 2.2.2. Dry Weight

##### Dry Weight of Leaves

The highest dry weight of leaves in the first season (9.71 g/plant) was recorded when plants were subjected to mineral fertilisation, compared to ½ CM + ½ RFL fertilisation (9.41 g/plant). Fertilisation with 100% CM produced reduced dry leaf weight (7.27 g/plant), which was equivalent to ⅓ CM + ⅓ RFL + AMF (6.68 g/plant), while the inoculated control generated the lowest weight (1.13 g/plant), comparable to the unfertilised control (1.18 g/plant).

In the subsequent season, the heaviest dry weight of leaves (3.31 g/plant) was achieved following fertilisation with 100% CM, comparable to ½ CM + ½ RFL (2.68 g/plant) and mineral (2.98 g/plant), outcompeting ⅓ CM + ⅓ RFL + AMF (1.78 g/plant). The lowest dry weight of leaves was attained from both unfertilised and inoculated controls (0.83 g/plant and 0.97 g/plant, respectively).

##### Dry Shoot Weight

Mineral fertilisation produced the highest dry shoot yield (21.25 g/plant), comparable to ½ CM + ½ RFL (17.61 g/plant), outperforming 100% CM (17.07 g/plant) and ⅓ CM + ⅓ RFL + AMF (14.24 g/plant) in the initial season. Both unfertilised and inoculated controls recorded the lowest dry shoot weight (2.27 g/plant and 2.12 g/plant, respectively).

In the succeeding season, the uppermost dry shoot yield (8.01 g/plant) was realised when plants were exposed to 100% CM fertilisation and was comparable to mineral fertilisation (7.04 g/plant) and ½ CM + ½ RFL fertilisation (5.84 g/plant), outweighing ⅓ CM + ⅓ RFL + AMF (3.89 g/plant). The unfertilised control recorded the lowest weight (1.80 g/plant) and was comparable to the inoculated control (2.03 g/plant).

##### Dry Root Weight

Plants fertilised in the first season with 100% CM, ½ CM + ½ RFL, and ⅓ CM + ⅓ RFL + AMF produced comparable dry root weight (ranging from 3.76 g/plant to 5.30 g/plant), outweighing both inoculated and unfertilised controls (0.49 g/plant and 0.62 g/plant, respectively). In the subsequent season, 100% CM fertiliser produced the highest dry root weight (2.02 g/plant), which was equivalent to mineral fertiliser (1.86 g/plant) and ½ CM + ½ RFL (1.46 g/plant), outweighing ⅓ CM + ⅓ RFL + AMF (0.97 g/plant). The lowest dry root weight was attained from the unfertilised and inoculated controls (0.37 g/plant and 0.48 g/plant, respectively).

### 2.3. Correlation Analysis

Table 2 shows the correlation between measured yield parameters and nutrient uptake in *Amaranthus* leaves following the treatment applications. In both cropping seasons, a strong positive correlation ranging from *r* = 0.841 to *r* = 0.971 in the first cropping season and *r* = 0.770 to r = 0.899 in the subsequent season was observed. Very significant correlations (**) were realised between the measured dry yield attributes and N, K, and Ca, while P and Mg only demonstrated significant interrelations (*) with the yield attributes in Season 1. In Season 2, a significant (*) correlation was attained between yield attributes and N, K, Ca, and Mg, while P had no significant relations with yield attributes.

### 2.4. Effects of Cattle Manure–Mineral Fertiliser Co-Fertilisation, and AMF Inoculation on Amaranthus Leaf Tissue Concentrations

Table 3 shows a significant increase in the concentrations of total N, P, Ca, and crude protein due to the application of cattle manure, mineral fertiliser, and AMF inoculation in both seasons. In the first season, fertilisation with 100% CM, ½ CM + ½ RFL, and ⅓ CM + ⅓ RFL + AMF, and RFL resulted in significantly higher leaf tissue concentrations of N, P, and Ca, as well as crude protein relative to both inoculated and unfertilised controls, which had the lowest concentrations of all the measured nutrients. No response was observed in the concentrations of K and Mg due to fertilisation or AMF inoculation. High concentrations of N and P were attained from *A. cruentus*, while no difference was observed between *Amaranthus* species in the concentration of other nutrients.

In the subsequent season, fertilisation with 100% CM, ⅓ CM + ⅓ RFL + AMF, and RFL recorded the highest concentrations of crude protein and total nitrogen, outcompeting ½ CM + ½ RFL. The highest P concentration was recorded in ⅓ CM + ⅓ RFL + AMF and in RFL compared with 100% CM and ½ CM + ½ RFL. Fertilisation with 100% CM, ½ CM + ½ RFL, ⅓ CM + ⅓ RFL + AMF, and RFL recorded improved and comparable concentrations of K, Ca, and Mg relative to both inoculated and unfertilised controls.

### 2.5. Nutrient Uptake by Leaves

Uptake of N, P, K, Ca, and Mg by leaves of *Amaranthus* species was significantly influenced by cattle manure and mineral fertiliser application coupled with AMF inoculation in both growing seasons (Table 4). In the first cropping season, the highest uptake of N, P, Ca, and Mg was recorded following fertilisation with mineral fertiliser and was comparable to that with 100% CM fertilisation, outperforming ½ CM + ½ RFL and ⅓ CM + ⅓ RFL + AMF. While fertilisation with ½ CM + ½ RFL and ⅓ CM + ⅓ RFL + AMF improved uptake of N, Ca, and Mg relative to unfertilised and inoculated controls, no influence was realised regarding uptake of P and K.

In the following cropping season, fertilisation with RFL resulted in the highest uptake of P, K, and Ca, which was comparable to 100% CM. The highest uptake of N and Mg was observed following 100% CM fertilisation, while similar performance for the uptake of N, P, K, Ca, and Mg was observed from ½ CM + ½ RFL and ⅓ CM + ⅓ RFL + AMF fertilisation. The lowest nutrient uptake was observed in both inoculated and unfertilised controls.

*A. cruentus* demonstrated the highest uptake of N, P, Ca, and Mg in the first season when compared to *A. hybridus*, and there were no significant differences realised in the following season.

### 2.6. Residual Soil Chemical Properties

In the first cropping season, the greatest improved pH (5.05) was recorded from RFL and comparable to both ⅓ CM + ⅓ RFL + AMF (4.96) and ½ CM + ½ RFL (4.79), outcompeting 100% CM (4.56) (Table 5). Both inoculated and unfertilised controls demonstrated the lowest pH, at 4.05 and 4.06, respectively. In the second cropping season, RFL also showed the most significant improvement in pH, surpassing ⅓ CM + ⅓ RFL + AMF (4.90) and ½ CM + ½ RFL (4.55), while the lowest and comparable pH was observed with AMF (4.15) and C (4.09).

In both cropping seasons, fertilisation with 100% CM, ½ CM + ½ RFL, ⅓ CM + ⅓ RFL + AMF, and RFL significantly decreased exchangeable acidity and acid saturation relative to the AMF-inoculated and unfertilised controls. Total cations significantly improved following fertilisation with RFL and outcompeted 100% CM, ½ CM + ½ RFL, and ⅓ CM + ⅓ RFL + AMF fertilisation, while the inoculated and unfertilised controls yielded the least.

### 2.7. Residual Soil Nutrients

Fertilisation with cattle manure and mineral fertiliser, in conjunction with AMF inoculation, significantly increased soil residual nutrients (Table 6). In the first cropping season, fertilisation with RFL resulted in the highest accumulation of soil K, Ca, Mg, and Zn, while the highest P accumulation was observed with ½ CM + ½ RFL, equivalent to RFL. Accumulation of soil nutrients where 100% CM, ½ CM + ½ RFL, and ⅓ CM + ⅓ RFL + AMF were applied, outweighed inoculated and unfertilised controls.

In Season 2, the highest accumulation of soil P was attained from RFL and was comparable to ½ CM + ½ RFL and ⅓ CM + ⅓ RFL + AMF. Fertilisation with RFL also resulted in the highest accumulation of K and Ca, while 100% CM resulted in the highest accumulation of Mg and Zn. Similar to the previous cropping season, inoculated and unfertilised controls produced the lowest accumulation of residual soil nutrients.

## 3. Discussion

Cattle manure and mineral fertiliser application, coupled with AMF inoculation, significantly improved vegetative growth, yield, leaf tissue nutrient composition, and nutrient uptake of both *Amaranthus* species compared to the inoculated and unfertilised controls across the growing seasons. This was also observed in soil residual nutrient composition and soil acidity-related properties.

Even though mineral fertilisation meaningfully triggered the highest plant height and number of leaves when relative to other fertilised treatments in the first cropping season, no significant differences were realised in the succeeding residual season, clarifying the fact that applied cattle manure, either alone or in combination, released nutrients slowly throughout the year of application which eventually did not meet the crop nutrient requirements as stated in [18].

Teke et al. [19] also reported improved plant height in garlic plants fertilised with animal manures, resulting from enhanced soil nitrogen. Similarly, Ref. [20] reported stimulated vegetative growth, increased leaf number and leaf area, and improved leaf fresh weight and quality of *Amaranthus* due to adequate soil nitrogen, which was responsible for both cell division and cell elongation in plants.

No significant differences were realised between 100% manure application, ½ CM + ½ RFL, and ⅓ CM + ⅓ RFL + AMF across the growing seasons, suggesting that manure application could be reduced by 50% and supplement the deficit with NPK fertiliser or decrease by 33% while complementing with 33% NPK and inoculating with AMF. The results are consistent with [21], which observed increased plant height and number of leaves in *Amaranthus* augmented with a combination of mycorrhizal fungi and compost relative to the unfertilised control.

Stem girth and root length were also induced by treatment application relative to both inoculated and unfertilised controls across the growing seasons. Teke et al. [19] observed that, among other benefits of applying cattle manure to the soil, is the improved soil texture, which enhances root production of the crop. In comparison, inoculating plants with AMF establishes a symbiosis with roots, thereby extending nutrient uptake beyond the normal root zone level through extended root elongation [16,22].

The development and expansion of the plant root system assist plants to adapt under stressful conditions of water and nutrient deficiencies, and this conforms to the findings of [20], which reported a significant increase in the stem girth and roots of *Amaranthus* following application of AMF inoculation with organic fertilisers compared to untreated plants.

The fresh and dry weight of *Amaranthus* leaves, shoots, and roots were also enhanced by application of treatments across the growing seasons. This, according to [23], further attests to the application of manures and mineral fertilisers in the soil, which, alone or in combination, improved soil nutrient status. This, in turn, increased the rate of plant photosynthesis, resulting in higher leaf quality and leaf weight. Application of treatments also increased leaf uptake of N, P, K, Ca, and Mg from the soil in *Amaranthus* species, which could translate into greater photosynthate accumulation, resulting in increased fresh and dry biomass.

This explains why plants exposed to 100% CM and ½ CM + ½ RFL produced biomass equivalent to RFL, while AMF, applied at the ⅓ CM + ⅓ RFL + AMF dose, promoted root growth and elongation, thereby improving plant performance. The poor performance of *Amaranthus* plants grown in both unfertilised and inoculated control plots indicates that a severe nutrient deficiency attained in acidic soils, probably due to adsorption, was a limiting factor for crop development [24]. This was further evidenced by significant positive correlations between nutrient uptake and both fresh and dry biomass in *Amaranthus*.

In the first season, the concentrations of N, P, Ca, Zn, and crude protein increased in the leaves of *Amaranthus* in response to treatment application, whereas no response was detected in K, Mg, and Fe. In the residual subsequent season, an improved concentration of crude protein, N, P, K, Ca, Mg, and Zn was realised except for Fe. Owuru et al. (2010) [25] reported that nutrients from the applied treatments were partitioned toward the vegetative parts of the plants and observed a direct proportionality between the applied rate of compost and the nutritional composition of *Amaranthus*. This is consistent with [16], which stated that nutrient concentrations in plant leaves usually vary with soil fertility, environment, plant species, and age. Furthermore, [20] reported enhanced nutrient concentration and nutrient uptake in leaves of *Amaranthus hybridus* fertilised with a combination of mycorrhiza and poultry manure.

Likewise, the application of treatments across the growing seasons improved residual soil chemical properties, namely pH, exchangeable acidity, total cations, and acid saturation. Similar advances were observed in residual soil nutrients (P, K, Ca, Mg, and Zn) following treatment application relative to inoculated and unfertilised controls in both seasons. This indicates that applying cattle manure improved the soil’s physical, chemical, and biological properties while also stimulating soil microbial activity, which could ultimately assist in plant nutrient release and healthy growth [21]. Post-cropping pH and total cations increased on plots where treatments were applied relative to both unfertilised and inoculated controls, while exchangeable acidity and acid saturation decreased considerably, implying that Ca^2+^ and Mg^2+^ ions in the applied treatments displaced H^+^, Fe^2+^, and Al^3+^ ions from soil adsorption sites, thereby increasing soil pH and total cations while decreasing the exchangeable acidity. Ameyu [26] reported the potential of manure or lime application to improve soil pH and total cation levels, while Ref. [21] observed enhanced availability of soil total N, available P, and total K on saline soils amended with combinations of biochar and AMF.

## 4. Materials and Methods

### 4.1. Study Area

#### 4.1.1. Location of the Study Area

**Figure 3:**
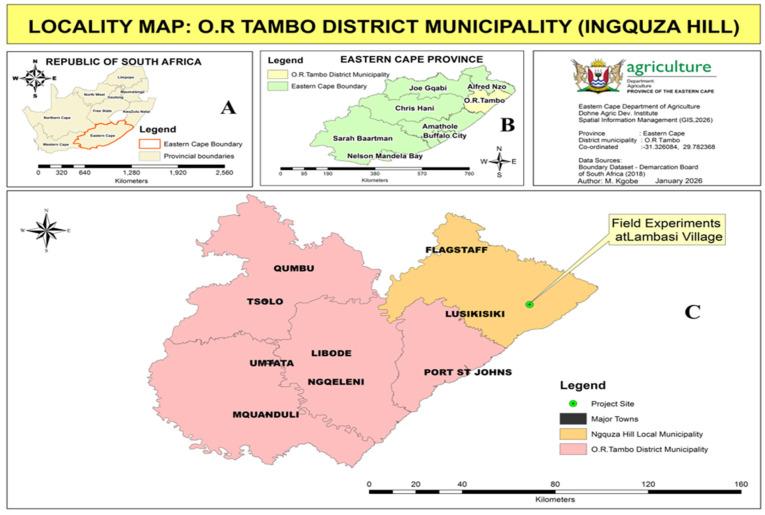
Locality map of the study area in Ingquza Hill Local Municipality, OR Tambo District, Eastern Cape: (**A**) South Africa, (**B**) Eastern Cape, and (**C**) OR Tambo District Municipality (Source: DRDAR-GIS).

Two (2) field experiments were conducted at Lambasi Village, Ingquza Hill Local Municipality, OR Tambo District, Eastern Cape Province, South Africa (Figure 3), for two consecutive seasons in 2020 and 2021. The area is located at −31.326084, 29.782368 (latitude and longitude) with an elevation of 475 m above sea level (ASL).

#### 4.1.2. Climatic Conditions

**Figure 4:**
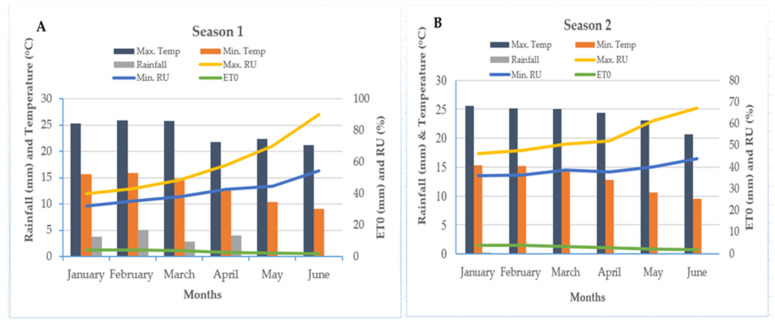
Climatic data (temperature, rainfall, relative humidity, and relative evapotranspiration) recorded at Ingquza Hill Local Municipality, OR Tambo District, in Season 1 (**A**) and Season 2 (**B**) (Source: Agricultural Research Council (ARC)—Climate & Weather Services). Max. temp = Maximum temperature; Min. temp = Minimum temperature; Max. RU = Maximum relative humidity; Min. RU = Minimum relative humidity; and ET0 = Total relative evapotranspiration.

The minimum temperature for both growing seasons ranged from 9 °C to 15 °C, while the maximum temperature ranged from 20 °C to 27 °C (Figure 4). For both minimum and maximum temperatures, the highest modes were achieved in January, February, and March, followed by a steady decline in April, May, and June.

While an average rainfall of 2 mm–5 mm was recorded between January and April in the first season, minimal rainfall, just above 0 mm, was observed in the subsequent season (Figure 4). A significant increase was observed in both maximum and minimum relative humidity (RH), followed by a general decrease in total relative evapotranspiration across the growing seasons.

#### 4.1.3. Soil Profile Description

The profile at the study site was characterised by an orthic A horizon over non-red stratified alluvium and a non-calcareous B horizon. Soils with such characteristics are classified as Dundee soil belonging to the Mtamvuna Family [27] and correspond well with Fluvisol in the World Reference Base (WRB) as per the description in [28].

### 4.2. Experimental Treatments

#### 4.2.1. Plant Material

Two *Amaranthus* species, namely *A. hybridus* and *A. cruentus,* were used as test crops in this study. *Amaranthus hybridus* is consumed as a leafy vegetable, while *A. cruentus* is regarded as a grain type *of Amaranthus* [29]. Seeds of *A. hybridus* were supplied by the Agricultural Research Council of South Africa (ARC), while *A. cruentus* was obtained from a reputable seed supplier, Ball Straathof (Pty) Ltd., based in Johannesburg, Gauteng Province, South Africa.

#### 4.2.2. Cattle Manure and Arbuscular Mycorrhizal Fungi (AMF)

Well-decomposed cattle manure used in the experiment was collected from a farmer’s kraal in Mlungisi Location, Amahlathi Local Municipality, Amathole District. At the same time, AMFs were sourced from Mycoroot (Pty) Ltd. at Rhodes University in Makhanda, Sarah Baartman District, Eastern Cape, South Africa. According to [30], each gram of Mycoroot AMF contains approximately 100 propagules, which have at least 10 spores per gram in four AMF species, namely *Glomus etunicatum*, *Paraglomus occultum*, *Glomus clarum*, and *Glomus mosseae*.

### 4.3. Sampling and Analysis of Soil and Cattle Manure Used in the Experiment

#### 4.3.1. Soil

Soil samples were collected from the experimental site before the experiment using a soil auger at a depth of 0–15 cm. Samples were analysed at the Döhne Agricultural Development Institute Analytical Laboratory to determine the soil’s physicochemical properties as well as fertiliser and lime requirements for growing *Amaranthus* species (Table 7).

Samples were air-dried, homogenised, ground, and sieved through a 2 mm sieve. Soil pH was measured in 1 mol KCl (potassium chloride) solution (pH_KCl_), and extractable phosphorus (P) was determined using the Bray-2 method as outlined in [31]. Organic carbon (OC), texture, and total nitrogen (N) were determined through the micro-Kjeldahl method, while potassium (K), calcium (Ca), and magnesium (Mg) were extracted following standard laboratory procedures outlined in [31].

#### 4.3.2. Cattle Manure

Manure samples were mixed to form homogenised representative samples before they were air-dried, ground, and sieved through a 2 mm sieve to remove larger materials before analysis. Samples were then analysed to determine their nutrient status and physicochemical properties, as shown in Table 7.

The pH of manure samples was measured in 1 mol KCl solution, while total nitrogen was analysed following the micro-Kjeldahl method. The phosphorus (P), potassium (K), calcium (Ca), magnesium (Mg), iron (Fe), copper (Cu), zinc (Zn), and manganese (Mn) were determined following the methods explained in [31].

### 4.4. Trial Establishment and Agronomic Practices

#### 4.4.1. Seedling Establishment

Seedlings of two *Amaranthus* species, *A. hybridus* and *A. cruentus*, were germinated and grown for five weeks in 200-hole polystyrene cavity trays using commercial growing medium, Hygromix. Healthy, uniform-sized seedlings were selected for field transplanting.

#### 4.4.2. Land Preparation and Transplanting

##### Land Preparation

The field was mechanically ploughed using a moldboard plough, disked and harrowed into a fine bed before it was manually marked out into plots measuring 3 m × 3 m (9 m^2^). Well-decomposed cattle manure and lime were broadcast and incorporated into the designated experimental plots a week later, while other treatments were applied during transplanting [32]. AMF inoculation together with manure and inorganic fertiliser treatments were administered in the first growing season (Season 1), while in the subsequent growing season (Season 2), the same plots were used without application of treatments and were regarded as a residual season.

##### Transplanting

Transplanting took place one month after applying the lime and cattle manure. Seedlings were transplanted at 30 cm intra-row on 50 cm inter-row spacing to give a plant population of 60 plants per plot (±66,667 plants/ha) [33].

Each gross plot consisted of six rows, and the four inner rows were used as the net plot. The two outer rows of the net plot were used to collect data on growth parameters, while the inner two were targeted for yield parameters [34]. After transplanting, the plots were manually irrigated using watering cans for the first few days to aid establishment. The plots were weeded manually using hand hoes to keep them weed-free throughout the study.

#### 4.4.3. Experimental Design and Layout

##### Treatment Combinations

Cattle manure application was based on the recommended rate of soil phosphorus (P) requirement (195 kg/ha) needed to satisfy the growth of *Amaranthus*. The same rate was considered when applying mineral (NPK) fertiliser in combination with 16.6 tons/ha of agricultural lime.

Manure was applied at three levels: 100%, 50%, and 33%. This resulted in three treatments and treatment combinations as follows: 100% cattle manure (CM), 50% CM + 50% NPK fertiliser and lime (½ CM + ½ NPK), and 33% cattle manure + 33% NPK and lime + AMF (⅓ CM + ⅓ NPK + AMF). Additionally, there were three further treatments: application of the recommended rate of mineral NPK fertiliser and lime (RFL) as a positive control, inoculation with arbuscular mycorrhizal fungi (AMF) only as an inoculated control, and an absolute control (C) with nothing applied.

In the AMF-inoculated treatments, seedlings were inoculated with AMF by placing 12 g of AMF granules per plant in each hole before transplanting, as per the rates applied in [35].

##### Design and Layout

The experiment consisted of two main plot treatments and six subplot treatments. The two *Amaranthus* species, *A. Hybridus* and *A. cruentus*, were the main plots, while amendment and application rate combinations were the subplots. It was then laid out as a factorial split-plot design, arranged in a randomised complete block design (RCBD) with three replications totalling 36 plots.

### 4.5. Data Collection

#### 4.5.1. Growth Parameters

Destructive sampling was performed, with two plants per plot being randomly selected and carefully uprooted from the net rows to determine growth parameters. The agronomic parameters measured for plant growth were plant height, number of leaves, stem girth, and root length, measured fortnightly at 2, 4, 6, and 8 weeks after transplanting (WAT), and the data were then pooled together. A metre (m) ruler was used to measure plant height as the vertical distance from the ground level to the apex of the meristem, expressed in centimetres (cm). The number of leaves was counted visually, while stem girth was measured as the diameter of the stem at its base using a Vernier calliper. Root length, expressed in centimetres (cm), was measured using a centimetre/millimetre ruler from the base of the stem to the longest root tip.

#### 4.5.2. Yield Parameters

The measured yield parameters were fresh and dry leaf weights, and fresh and dry shoot yields. Freshly harvested leaves and fresh shoot yield were weighed on the day of harvesting using an Adam CBW-3 scale to account for fresh weight yield. Dry weight of leaves and dry shoot yield were determined using the same scale after oven-drying at 65 °C as a constant weight to account for dry weight yield [21]. The percentage increase in growth and yield was calculated using the formula from [36]:

(1) $$Increase\;in\;yield\left(\%\right)=\frac{Yield\;of\;treatment-Yield\;of\;control}{Yield\;of\;control}\times 100\;$$

#### 4.5.3. Nutrient Analysis

Four (4) random plants per plot during final harvesting (i.e., at 8 WAT) were harvested for the determination of nutrient concentration in leaves as well as nutrient uptake. Leaves of the harvested plants were dried in an oven at 65 °C to constant weight as described in [21]. All oven-dried leaf samples were ground, digested, and analysed to determine the concentrations of total N, P, K, Ca, and Mg in the leaves of *Amaranthus* as described in [37].

Nutrient uptake in plants was determined by multiplying the nutrient concentration in the plant samples by the dry matter yield [37], which was calculated using the following formula:

(2) $$Nutrient\;uptake(mg/plant)=\frac{Nutrient\;content\left(\%\right)\;\times \;Yield\;(mg/plant)}{100}$$

### 4.6. Statistical Analysis

All collected data were subjected to statistical analysis as per [38]. Analysis of variance (ANOVA) followed by least significant difference (LSD) was used to test differences among treatments. Where treatment means were found to be significant, Duncan’s multiple range test (DMRT) was used to separate them at *p* ≤ 0.05. Correlations between the measured treatments were determined using Pearson’s correlation coefficients.

## 5. Conclusions

Inoculation of *Amaranthus* plants grown in acidic soil with AMF alone had no significant effect on growth, yield, leaf tissue nutrient concentrations, and nutrient uptake across the growing seasons. This suggests that under stressful conditions of limited nutrient supply to minimally mycorrhizal plants such as *Amaranthus* species, AMF contribution towards nutrient availability could be insufficient. On the contrary, improved agronomic performance of *Amaranthus* species was observed following inoculation with AMF in conjunction with 33% CM and 33% RFL, demonstrating the benefits of such inoculation even with low nutrient additions.

Although application of a mixture of cattle manure and mineral fertiliser, as well as sole cattle manure, also improved *Amaranthus* performance to be on par with mineral fertilisation, bulkiness and low P content in cattle manure necessitated very high application rates to achieve yields comparable to mineral fertilisation. As a result, smallholder farmers in the Eastern Cape apply insufficient amounts of fertiliser. However, these farmers cannot afford mineral fertilisers due to their limited financial resources. The results of the present study suggest that applying a third of cattle manure, coupled with a third of mineral fertilisers inoculated with AMF, could improve the yields of farmers producing crops in acidic soils.

## Figures and Tables

**Table 1:** Effects of cattle manure–mineral fertiliser co-fertilisation and AMF inoculation on growth parameters of *Amaranthus*.

		Growth Parameters			
		Plant Height	Number of Leaves	Stem Girth	Root Length
		cm plant^−1^	plant^−1^	cm plant^−1^	
Season	Treatments				
Season 1	C	30.72 ± 3.1 ^a^	26.42 ± 4.4 ^a^	5.35 ± 0.7 ^a^	11.81 ± 0.9 ^a^
	AMF	31.61 ± 2.8 ^a^	27.69 ± 3.0 ^a^	5.74 ± 0.3 ^a^	12.15 ± 0.4 ^a^
	CM	61.36 ± 3.4 ^b^	75.12 ± 12.7 ^b^	12.30 ± 0.6 ^b^	17.42 ± 0.5 ^b^
	½ CM + ½ RFL	65.23 ± 2.8 ^b^	75.65 ± 18.2 ^b^	13.42 ± 0.8 ^bc^	17.44 ± 1.1 ^b^
	⅓ CM + ⅓ RFL + AMF	66.05 ± 1.4 ^b^	82.00 ± 14.0 ^bc^	13.96 ± 0.7 ^bc^	17.87 ± 0.8 ^b^
	RFL	74.39 ± 4.2 ^c^	93.00 ± 18.6 ^c^	15.33 ± 0.8 ^c^	18.32 ± 0.5 ^b^
	LSD	7.71	13.72	1.96	2.04
	*p* Value	<0.001	<0.001	<0.001	<0.001
	*Amaranthus* species				
	*A. hybridus* L.	52.36 ± 4.59 ^a^	87.73 ± 10.1 ^b^	10.85 ± 1.0 ^a^	15.61 ± 0.8 ^a^
	*A. cruentus* L.	57.44 ± 4.30 ^a^	38.91 ± 3.6 ^a^	11.19 ± 1.0 ^a^	16.05 ± 0.8 ^a^
	LSD	10.59	23.09	1.33	2.35
	*p* Value	0.033	<0.001	0.562	0.501
Season 2	C	24.95 ± 2.6 ^a^	21.83 ± 2.7 ^a^	4.87 ± 0.3 ^a^	9.34 ± 0.4 ^a^
	AMF	25.63 ± 2.4 ^a^	22.85 ± 3.0 ^a^	5.16 ± 0.2 ^a^	9.85 ± 0.4 ^a^
	CM	50.57 ± 5.6 ^c^	45.46 ± 9.8 ^b^	9.23 ± 0.9 ^c^	13.33 ± 0.7 ^c^
	½ CM + ½ RFL	42.35 ± 5.5 ^bc^	42.42 ± 10.6 ^b^	8.16 ± 0.8 ^bc^	12.13 ± 0.7 ^bc^
	⅓ CM + ⅓ RFL + AMF	36.69 ± 3.4 ^b^	36.17 ± 7.6 ^b^	7.06 ± 0.4 ^b^	12.78 ± 0.3 ^bc^
	RFL	47.46 ± 5.4 ^c^	46.25 ± 8.4 ^b^	9.41 ± 0.7 ^c^	12.73 ± 0.9 ^bc^
	LSD	9.18	11.86	1.38	1.43
	*p* Value	<0.001	<0.001	<0.001	<0.001
	*Amaranthus* species				
	*A. hybridus* L.	44.74 ± 2.8 ^a^	48.86 ± 4.8 ^b^	7.96 ± 0.5 ^a^	11.25 ± 0.5 ^a^
	*A. cruentus* L.	31.15 ± 3.1 ^b^	22.80 ± 1.8 ^a^	6.67 ± 0.6 ^a^	12.14 ± 0.4 ^a^
	LSD	3.38	20.64	1.39	2.05
	*p* Value	<0.001	0.032	0.063	0.204

Different superscript letters (a, b, and c) within a column indicate differences between treatment combinations.

**Table 2:** Pearson correlation coefficients between the measured yield parameters and nutrient uptake by *Amaranthus* leaves.

Season 1								
**Variable**	**DLW**	**DSY**	**DRW**	**N**	**P**	**K**	**Ca**	**Mg**
DLW	—							
DSY	0.996 ***	—						
DRW	0.987 ***	0.996 ***	—					
N	0.900 **	0.920 **	0.944 **	—				
P	0.841 *	0.857 *	0.893 *	0.977 ***	—			
K	0.900 **	0.926 **	0.952 **	0.994 ***	0.962 **	—		
Ca	0.931 **	0.957 **	0.971 **	0.971 **	0.908 **	0.986 ***	—	
Mg	0.859 *	0.894 *	0.921 *	0.97 **	0.915 **	0.987 ***	0.985 ***	—
Season 2								
DLW	—							
DSY	0.996 ***	—						
DRW	0.995 ***	0.999 ***	—					
N	0.890 *	0.885 *	0.899 *	—				
P	0.770 ^ns^	0.753 ^ns^	0.775 ^ns^	0.967 **	—			
K	0.821 *	0.812 *	0.83 *	0.989 ***	0.993 ***	—		
Ca	0.818 *	0.813 *	0.826 *	0.987 ***	0.973 **	0.99 ***	—	
Mg	0.890 *	0.890 *	0.894 *	0.976 ***	0.909 **	0.949 **	0.976 ***	—

Correlation is highly significant at the 0.1% level (***); very significant at the 1.0% level (**); significant at the 5.0% level (*); and non-significant (ns), respectively. DLW = Dry Weight of Leaves, DSY = Dry Shoot Yield, DRW = Dry Weight of Roots, N = Nitrogen, P = Phosphorus, K = Potassium, Ca = Calcium, and Mg = Magnesium.

**Table 3:** Effects of cattle manure–mineral fertiliser co-fertilisation and AMF inoculation on leaf nutrient concentration of *Amaranthus*.

	Nutrient Concentration in Leaves of *Amaranthus* Species					
	Crude Protein	Total N	P	K	Ca	Mg
	%	g/100 g				
Season 1						
**Treatments**						
C	15.08 ^a^	1.86 ^a^	0.18 ^a^	0.95 ^a^	1.84 ^a^	0.87 ^a^
AMF	15.08 ^a^	1.91 ^a^	0.27 ^ab^	1.00 ^a^	2.01 ^a^	1.06 ^a^
CM	26.62 ^b^	4.16 ^b^	0.76 ^abc^	1.03 ^a^	3.79 ^b^	1.38 ^a^
½ CM + ½ RFL	23.80 ^ab^	3.54 ^ab^	0.60 ^abc^	0.93 ^a^	4.00 ^b^	1.23 ^a^
⅓ CM + ⅓ RFL + AMF	27.20 ^b^	4.35 ^b^	0.87 ^bc^	1.00 ^a^	4.27 ^b^	1.34 ^a^
RFL	32.53 ^b^	4.94 ^b^	1.00 ^c^	1.26 ^a^	3.87 ^b^	1.24 ^a^
LSD	7.4	1.15	0.45	0.4	1.14	0.39
*p* Value	<0.001	<0.001	<0.001	0.59	<0.001	0.1
*Amaranthus* species						
*A. hybridus* L.	19.21 ^a^	2.59 ^a^	0.41 ^a^	1.01 ^a^	2.88 ^a^	1.03 ^a^
*A. cruentus* L.	27.56 ^a^	4.33 ^b^	0.81 ^b^	1.04 ^a^	3.72 ^a^	1.34 ^a^
LSD	13.25	1.17	0.18	0.34	1.1	0.33
*p* Value	0.11	0.03	<0.001	0.73	0.08	0.05
Season 2						
**Treatments**						
C	10.31 ^a^	1.65 ^a^	0.08 ^a^	0.44 ^a^	3.82 ^a^	1.41 ^a^
AMF	11.04 ^a^	1.77 ^a^	0.09 ^a^	0.43 ^a^	4.13 ^a^	1.37 ^a^
CM	27.01 ^bc^	4.32 ^bc^	0.13 ^ab^	0.66 ^ab^	7.18 ^b^	2.33 ^b^
½ CM + ½ RFL	23.73 ^b^	3.80 ^b^	0.15 ^b^	0.84 ^b^	7.99 ^b^	1.89 ^b^
⅓ CM + ⅓ RFL + AMF	26.44 ^bc^	4.23 ^bc^	0.19 ^c^	0.91 ^b^	7.19 ^b^	1.92 ^b^
RFL	29.88 ^c^	4.78 ^c^	0.20 ^c^	0.98 ^b^	8.52 ^b^	1.80 ^ab^
LSD	4.4	0.7	0.05	0.35	1.77	0.5
*p* Value	<0.001	<0.001	<0.001	0.01	<0.001	0.01
*Amaranthus* species						
*A. hybridus* L.	21.39 ^a^	3.42 ^a^	0.13 ^a^	0.66 ^a^	5.87 ^a^	1.77 ^a^
*A. cruentus* L.	21.41 ^a^	3.43 ^a^	0.15 ^a^	0.76 ^a^	7.07 ^a^	1.80 ^a^
LSD	5.84	0.93	0.14	0.59	3.04	0.75
*p* Value	0.99	0.99	0.54	0.56	0.23	0.89

Different superscript letters (a, b, and c) within a column indicate differences between amendments/treatment combinations.

**Table 4:** Effects of cattle manure–mineral fertiliser co-fertilisation and AMF inoculation on leaf nutrient uptake by *Amaranthus* leaves.

		Nutrient Uptake by Leaves of *Amaranthus* Species				
		N	P	K	Ca	Mg
		mg kg^−1^				
Season	Treatments					
Season 1	C	42.31 ± 11.4 ^a^	21.83 ± 0.9 ^a^	19.32 ± 3.6 ^a^	35.40 ± 7.1 ^a^	1.58 ± 0.3 ^a^
	AMF	47.64 ± 14.3 ^a^	21.86 ± 0.7 ^a^	20.83 ± 3.5 ^a^	42.31 ± 7.3 ^a^	2.14 ± 0.3 ^a^
	CM	291.72 ± 55.7 ^c^	63.77 ± 16.8 ^bc^	90.04 ± 33.5 ^b^	242.33 ± 42.8 ^bc^	12.14 ± 3.2 ^c^
	½ CM + ½ RFL	206.52 ± 44.6 ^b^	41.75 ± 9.5 ^a^	54.73 ± 12.0 ^ab^	226.15 ± 47.2 ^bc^	7.41 ± 1.7 ^b^
	⅓ CM + ⅓ RFL + AMF	180.53 ± 41.9 ^b^	40.17 ± 7.8 ^a^	37.75 ± 3.8 ^a^	167.83 ± 28.6 ^b^	5.37 ± 1.2 ^ab^
	RFL	306.15 ± 47.9 ^c^	72.37 ± 15.0 ^c^	92.99 ± 25.7 ^b^	253.08 ± 43.3 ^c^	8.95 ± 2.4 ^bc^
	LSD	77.96	23.52	46.93	76.44	4.29
	*p* Value	<0.001	<0.001	0.009	<0.001	<0.001
	*Amaranthus* species					
	*A. hybridus* L.	124.80 ± 25.5 ^a^	31.26 ± 3.9 ^a^	47.65 ± 13.5 ^a^	114.78 ± 20.5 ^a^	4.47 ± 1.0 ^a^
	*A. cruentus* L.	233.43 ± 34. 9 ^b^	55.99 ± 8.6 ^b^	57.56 ± 10.7 ^a^	207.56 ± 31.1 ^b^	8.06 ± 1.5 ^b^
	LSD	35.41	5.57	49.93	52.07	2.60
	*p* Value	0.006	0.003	0.820	0.017	0.027
Season 2	C	29.42 ± 4. 8 ^a^	1.47 ± 0.5 ^a^	7.17 ± 1.9 ^a^	68.24 ± 12.8 ^a^	25.17 ± 2.9 ^a^
	AMF	35.64 ± 4.5 ^a^	1.83 ± 0.6 ^a^	8.42 ± 2.2 ^a^	84.53 ± 17.7 ^a^	27.27 ± 3.0 ^a^
	CM	344.35 ± 65.6 ^c^	10.78 ± 2.9 ^bc^	53.27 ± 15.5 ^bc^	550.32 ± 92.9 ^c^	193.58 ± 44.1 ^c^
	½ CM + ½ RFL	224.37 ± 50.8 ^b^	8.47 ± 1.7 ^b^	45.30 ± 9.0 ^bc^	456.41 ± 90.0 ^bc^	116.94 ± 30.7 ^b^
	⅓ CM + ⅓ RFL + AMF	168.63 ± 28.1 ^b^	7.65 ± 2.0 ^b^	37.44 ± 12.5 ^b^	286.37 ± 51.2 ^b^	77.57 ± 16.2 ^ab^
	RFL	339.44 ± 160.6 ^c^	13.82 ± 2.1 ^c^	65.72 ± 7.2 ^c^	589.58 ± 94.7 ^c^	125.40 ± 16.8 ^b^
	LSD	113.73	4.26	21.57	202.37	66.48
	*p* Value	<0.001	<0.001	<0.001	<0.001	<0.001
	*Amaranthus* species					
	*A. hybridus* L.	196.62 ± 41.3 ^a^	6.68 ± 1.36 ^a^	35.38 ± 8.0 ^a^	332.55 ± 71.1 ^a^	94.65 ± 19.3 ^a^
	*A. cruentus* L.	183.95 ± 34.5 ^a^	8.00 ± 1.55 ^a^	37.06 ± 6.8 ^a^	345.81 ± 53.8 ^a^	94.00 ± 19.4 ^a^
	LSD	332.16	14.60	28.48	440.28	199.43
	*p* Value	0.890	0.743	0.821	0.909	0.990

Different superscript letters (a, b, and c) within a column indicate differences between amendments/treatment combinations.

**Table 5:** Effects of cattle manure–mineral fertiliser co-fertilisation and AMF inoculation on selected residual soil acidity-related properties.

		Selected Residual Soil Chemical Properties			
		pH	Exchangeable Acidity	Total Cations	Acid Saturation
			cmol L^−1^		%
Season	Treatments				
Season 1	C	4.06 ± 0.0 ^a^	1.68 ± 0.4 ^b^	5.51 ± 2.0 ^a^	15.00 ± 3.5 ^b^
	AMF	4.05 ± 0.0 ^a^	1.59 ± 0.4 ^b^	5.83 ± 1.3 ^a^	17.83 ± 4.9 ^b^
	CM	4.56 ± 0.1 ^ab^	0.28 ± 0.1 ^a^	7.38 ± 0.4 ^a^	3.50 ± 0.6 ^a^
	½ CM + ½ RFL	4.79 ± 0.2 ^bc^	0.60 ± 0.0 ^a^	9.45 ± 0.6 ^b^	6.33 ± 1.0 ^a^
	⅓ CM + ⅓ RFL + AMF	4.96 ± 0.3 ^bc^	0.68 ± 0.2 ^a^	8.78 ± 2.3 ^b^	8.83 ± 1.6 ^ab^
	RFL	5.05 ± 0.3 ^c^	0.19 ± 0.0 ^a^	17.52 ± 1.0 ^c^	1.83 ± 0.8 ^a^
	LSD	0.55	0.722	3.327	8.19
	*p* Value	0.002	<0.001	<0.001	0.003
Season 2	C	4.09 ± 0.0 ^a^	1.35 ± 0.0 ^b^	5.52 ± 0.4 ^a^	26.33 ± 1.9 ^b^
	AMF	4.15 ± 0.1 ^a^	1.51 ± 0.1 ^b^	6.54 ± 0.8 ^a^	24.17 ± 5.2 ^b^
	CM	4.51 ± 0.1 ^b^	0.54 ± 0.1 ^a^	7.34 ± 0.2 ^b^	6.17 ± 0.7 ^a^
	½ CM + ½ RFL	4.55 ± 0.1 ^b^	0.78 ± 0.1 ^a^	6.64 ± 0.1 ^a^	7.50 ± 1.0 ^a^
	⅓ CM + ⅓ RFL + AMF	4.90 ± 0.2 ^b^	1.15 ± 0.2 ^b^	8.65 ± 0.3 ^b^	12.17 ± 6.8 ^a^
	RFL	5.61 ± 0.4 ^c^	0.20 ± 0.4 ^a^	10.05 ± 0.6 ^c^	5.33 ± 3.0 ^a^
	LSD	0.32	0.892	1.157	10.77
	*p* Value	<0.001	0.047	<0.001	<0.001

Different superscript letters (a, b, and c) within a column indicate differences between amendments/treatment combinations.

**Table 6:** Effects of cattle manure–mineral fertiliser co-fertilisation and AMF inoculation on selected residual soil nutrients.

		Selected Residual Soil Nutrients				
		P	K	Ca	Mg	Zn
		mg kg^−1^				
Season	Treatments					
Season 1	C	23.33 ± 4.8 ^a^	236.71 ± 13.0 ^a^	374.08 ± 69.9 ^a^	172.22 ± 25.4 ^a^	1.35 ± 0.4 ^a^
	AMF	35.01 ± 5.1 ^a^	212.86 ± 10.6 ^a^	363.16 ± 65.5 ^a^	140.58 ± 82.3 ^a^	1.03 ± 0.2 ^a^
	CM	88.23 ± 5.7 ^a^	553.85 ± 99.8 ^b^	856.76 ± 45.0 ^b^	172.26 ± 25.5 ^a^	8.03 ± 0.7 ^b^
	½ CM + ½ RFL	256.34 ± 9.8 ^c^	513.87 ± 69.6 ^b^	1363.09 ± 137.6 ^c^	376.84 ± 161.6 ^a^	16.17 ± 4.8 ^cd^
	⅓ CM + ⅓ RFL + AMF	184.36 ± 26.5 ^b^	482.86 ± 21.6 ^b^	888.98 ± 239.3 ^b^	330.29 ± 112.4 ^a^	12.32 ± 2.9 ^c^
	RFL	213.32 ± 43.0 ^bc^	828.74 ± 195.0 ^c^	2401.75 ± 314.7 ^d^	662.76 ± 136.0 ^b^	27.45 ± 6.8 ^d^
	LSD	67.41	236.34	314.37	249.46	7.41
	*p* Value	<0.001	<0.001	<0.001	0.005	<0.001
Season 2	C	16.33 ± 4.3 ^a^	39.52 ± 7.7 ^a^	221.76 ± 35.7 ^a^	361.05 ± 24.5 ^a^	1.83 ± 0.6 ^ab^
	AMF	19.83 ± 4.7 ^a^	44.53 ± 8.1 ^a^	288.54 ± 36.9 ^a^	394.42 ± 14.9 ^ab^	1.13 ± 0.3 ^a^
	CM	33.00 ± 1.4 ^b^	152.56 ± 31.7 ^c^	482.53 ± 27.9 ^b^	490.38 ± 6.6 ^c^	5.07 ± 0.6 ^c^
	½ CM + ½ RFL	40.50 ± 5.1 ^c^	98.72 ± 5.1 ^b^	415.86 ± 31.3 ^b^	424.87 ± 24.0 ^b^	2.97 ± 0.2 ^b^
	⅓ CM + ⅓ RFL + AMF	35.67 ± 2.3 ^bc^	104.04 ± 15.3 ^b^	781.89 ± 111.2 ^c^	400.81± 13.9 ^a^	3.22 ± 0.3 ^b^
	RFL	63.17 ± 3.0 ^d^	164.84 ± 45.7 ^c^	1148.54 ± 144.9 ^d^	419.85 ± 20.8 ^b^	5.05 ± 0.6 ^c^
	LSD	7.32	48.88	173.13	40.56	1.18
	*p* Value	<0.001	<0.001	<0.001	<0.001	<0.001

Different superscript letters (a, b, c, and d) within a column indicate differences between amendments/treatment combinations.

**Table 7:** Physicochemical properties of soils and animal manures used in this study, as well as lime and nutrient requirements of *Amaranthus* species.

Soil		Cattle Manure	
Properties	Value	Properties	Value
pH (KCl)	3.99	pH (KCl)	7.75
Acid saturation (%)	55		
P (g kg^−1^)	1.1	Macro-nutrients (g kg^−1^)	
Exch. acidity (Cmol L^−1^)	3.75	Total N	10
Organic carbon (%)	1.97	P	0.9
Total cation (Cmol L^−1^)	6.83	Ca	8.8
* PAS (%)	5	Mg	6.8
N required (kg ha^−1^)	100	K	13.9
P required (kg ha^−1^)	195		
K required (kg ha^−1^)	365	Micro-nutrients (mg kg^−1^)	
Lime required (tons ha^−1^)	16.5	Na	186
		Cu	161
Exchangeable bases (g kg^−1^)		Fe	6805
K	5.4	Mn	440
Ca	41.8	Zn	108
Mg	10.4		
		Crude protein (%)	0.5
Texture analysis		Organic matter (%)	39.12
Sand (%)	76.6	Ash (%)	60.88
Silt (%)	7		
Clay (%)	16		
Textural class	Sandy loam		
Soil form	Dundee		
Accessible moisture (mm)	106		
Infiltration rate (mm h^−1^)	7		

* PAS = permissible acid saturation.

## Data Availability

The authors confirm that the data supporting the findings of this study are available within the article.

## Abbreviations

The following abbreviations are used in this manuscript:

P	Phosphorus	H_2_O	Water
Al	Aluminium	°C	Degrees Celsius
Fe	Iron	Mn	Manganese
AMF	Arbuscular mycorrhizal fungi	CM	Cattle manure
N	Nitrogen	C	Control
K	Potassium	cm	Centimetre
Ca	Calcium	g	Gram
Mg	Magnesium	%	Per cent
Zn	Zinc	kg	Kilogram
NPK	Mineral fertiliser	ARC	Agricultural Research Council
RFL	Recommended mineral fertiliser	OC	Organic carbon
Ca^2+^	Calcium ions	RCBD	Randomised complete block design
CaOH_2_	Calcium hydroxide	WAT	Weeks after transplanting
Al^3+^	Aluminium ions	m	Metre
(OH)_3_	Insoluble aluminium	LSD	Least significant difference
H^+^	Hydrogen ions	DMRT	Duncan multiple range test
Ca(OH)_2_	Calcium hydroxide	Cu	Copper
DRDAR	Department of Rural Development and Agrarian Reform	PAS	Permissible acid saturation
Pty	Proprietary		
Ltd	Limited		
